# A multi-mineral intervention is associated with improved intestinal permeability in patients with ulcerative colitis: results from a pilot trial

**DOI:** 10.3389/fmed.2026.1805900

**Published:** 2026-06-22

**Authors:** Muhammad N. Aslam, Danielle Kim Turgeon, Shannon McClintock, Ron Allen, Ananda Sen, James Varani

**Affiliations:** 1 Department of Pathology, University of Michigan Medical School, Ann Arbor, MI, United States; 2 Department of Internal Medicine, University of Michigan Medical School, Ann Arbor, MI, United States; 3 Department of Family Medicine, University of Michigan Medical School, Ann Arbor, MI, United States; 4 Department of Biostatistics, University of Michigan Medical School, Ann Arbor, MI, United States

**Keywords:** Aquamin, gut barrier, inflammatory bowel disease, intestinal permeability, minerals, trace elements, ulcerative colitis

## Abstract

**Introduction:**

Previous studies have shown that Aquamin^®^, a multi-mineral extract from red marine algae, enhances gut barrier protein expression in the human colon. Here we carried out an exploratory study aimed at generating support for Aquamin^®^ ’s ability to affect gastrointestinal permeability.

**Methods:**

Subjects with mild ulcerative colitis (UC) or UC in remission and healthy controls were enrolled in an open-label, pilot-phase trial and received Aquamin^®^ capsules (800 mg calcium/day) for 90 days. Intestinal permeability was evaluated before and after the 90-day intervention by urinary mannitol excretion after ingestion of a 5 g mannitol solution, with collections across several time intervals (pre-drink, 0–2 h, 2–8 h, and 8–24 h). The primary outcome was the change in mannitol excretion. Serum samples were also collected to assess liver and renal function.

**Results:**

In this pilot study (NCT04855799), which included UC patients and healthy controls (*n* = 8 per group), baseline urine mannitol levels in the 0–2 h sample were 54% higher in UC patients compared to healthy subjects (*p* = 0.006). Following 90 days of Aquamin^®^ supplementation, urinary mannitol levels in UC patients decreased by 28, 26, and 41% at the 0–2 h, 2–8 h, and 8–24 h time points, respectively; the reduction at the 0–2 h interval reached statistical significance (*p* = 0.015). Overall, Aquamin^®^ supplementation reduced total post-intervention mannitol excretion by 29% (*p* = 0.024). Aquamin^®^ was well tolerated, with no serious adverse events reported. Consistent with our earlier findings, the serum metabolic panel revealed a modest but statistically significant reduction in alkaline phosphatase levels after 90 days of intervention.

**Conclusion:**

Results from this exploratory study provide preliminary evidence that Aquamin^®^ supplementation is associated with improved gut barrier function in UC patients. These findings support further investigation of Aquamin^®^ as a safe and promising adjunct to current UC management strategies, with potential utility as a barrier therapy in UC.

**Clinical trial registration:**

https://clinicaltrials.gov/study/NCT04855799, identifier NCT04855799.

## Introduction

Inflammatory bowel disease (IBD), which includes ulcerative colitis (UC) and Crohn’s disease (CD), is characterized by chronic mucosal inflammation linked to impaired intestinal barrier function and increased permeability, or “leaky gut” (1–3). Normally, the epithelial barrier maintains selective absorption and defends against tissue influx of luminal pathogens, antigens, and toxins. In IBD, this barrier breaks down, allowing translocation of microbes and antigens into the submucosa, to fuel immune activation and ongoing inflammation. Notably, barrier defects may precede and perpetuate mucosal inflammation, acting as both cause and consequence of disease (4–8). In UC, diffuse colonic inflammation and superficial ulcers correspond with barrier dysfunction across disease severities. Even patients in remission can have persistent symptoms associated with ongoing permeability defects, which correlate with relapse risk and response to therapy (1–3). Thus, assessment of intestinal permeability is increasingly used for disease monitoring and management in UC.

Given this central pathogenic role, interest has grown in therapies aimed at restoring epithelial integrity and improving barrier function in UC. Numerous micronutrients, traditional remedies and natural product supplements have been evaluated for their ability to improve permeability control and support gut barrier health (2, 9). Despite encouraging results in experimental models and preliminary human studies, however no intervention has yet to demonstrate sufficient efficacy needed to justify widespread adoption (2).

For the past several years, our laboratory has investigated the potential of a calcium-rich, magnesium-rich, multi-mineral product known as Aquamin^®^ to support gastrointestinal (GI) health. In long-term (15–18 month) interventional studies in mice, inclusion of Aquamin^®^ in the diet suppressed polyp formation throughout the GI tract (10, 11) as well as liver tumor formation (12). A reduction in GI and systemic inflammation was seen in conjunction with these health benefits (10–16). Consistent with evidence of anti-inflammatory potential, Aquamin^®^ has also demonstrated the ability to suppress colitis development in the IL-10^–/–^ mouse model (17).

How the multi-mineral product promotes GI health is not fully understood. Studies using human colonic tissue in organoid culture (derived from both healthy individuals and from UC patients) have demonstrated increased elaboration of multiple proteins that contribute to barrier structure/function (18–22). An increase in trans-epithelial electrical resistance (TEER) and enhanced organoid cohesion accompanied these protein changes (20–22). Barrier proteins remained upregulated with Aquamin^®^ even when the colon organoids were challenged with a mix of LPS and three pro-inflammatory cytokines (22, 23). Most importantly, many of the same barrier protein changes observed in human colon tissue-derived organoid cultures were also observed in colon biopsies obtained from healthy subjects following daily ingestion of Aquamin^®^ over a 90-day period (24).

Based on the findings presented above, we carried out a 180-day interventional trial in patients with UC in remission or with mild disease (ClinicalTrials.gov ID: NCT03869905) (25). The goal of the trial was to determine if disease-related and mechanistic biomarkers could be improved in this population with Aquamin^®^. Over the 180-day treatment period, we saw reductions in several disease-related biomarkers that are typically elevated in UC. Among these were serum C-reactive protein (CRP), serum total and intestinal alkaline phosphatase (ALP), and fecal calprotectin. Histological evidence of reduced colonic inflammation was also seen. These changes were not observed in patients receiving placebo. Colon tissue biopsies obtained at the end of the interventional period also demonstrated increased expression of several gut barrier proteins compared to pre-intervention levels in the Aquamin^®^-treated cohort. These changes were not observed with placebo. The results from the interventional study allow us to suggest that Aquamin^®^ could serve as part of a maintenance regimen for individuals with mild UC or UC in remission (25).

The findings presented in our interventional trial along with the preclinical findings also allow us to hypothesize improvement in gastrointestinal barrier function as (at least) part of Aquamin^®^’s mechanism of action. To date, however, there is no direct evidence demonstrating improved barrier function *in vivo*. As a way to address this issue, mannitol recovery in the urine over a 24-h period following ingestion of the probe was assessed in a cohort of subjects (8 healthy individuals and 8 subjects with mild UC or UC in remission) before and after 90 days of Aquamin^®^ treatment under the same conditions used in the recent clinical study described above (25). The results of this study are described here.

## Materials and methods

### Intervention (Aquamin^®^)

This open-label interventional study utilized Aquamin^®^ following its designation as an Investigational New Drug (IND# 141600) by the U.S. Food and Drug Administration (FDA). Aquamin^®^ is a product rich in calcium, magnesium, and numerous trace minerals, and is sourced from calcified fronds of marine red algae belonging to the *Lithothamnion* genus (26). The formulation contains calcium and magnesium in approximately a 12:1 ratio, along with measurable amounts of seventy-two additional trace elements. Marketed as a dietary supplement (GRAS 000028; Marigot Ltd., Cork, Ireland), Aquamin^®^ is incorporated into foods and supplements available in Europe, Asia, Australia, and North America. Aquamin^®^ is available in multiple different preparations; for this trial, a single batch of Aquamin-TG^®^ (Food Grade) was encapsulated in hydroxypropyl methylcellulose (HPMC). Each capsule contained 600 mg of Aquamin^®^ standardized to deliver 200 mg elemental calcium. The University of Michigan Research Pharmacy dispensed a 90-day supply of Aquamin^®^ to participants. Each participant took four capsules per day, two in the morning and two in the evening, to achieve a total daily intake of 800 mg calcium, administered in addition to their ongoing UC maintenance therapy. The full elemental profile and daily intake amounts (of Aquamin-TG^®^) are presented in Supplementary Table 1. The mineral content of Aquamin-TG^®^ was verified by independent analysis (Advanced Laboratories, Salt Lake City, Utah) using Inductively Coupled Plasma Optical Emission Spectrometry; this intervention has been utilized in previous clinical research (24, 25, 27).

### Study design

This single-site study was a pilot-phase, open-label interventional trial that included participants with UC, either in remission or with mild disease, as well as healthy individuals. The primary objective was to determine whether daily Aquamin^®^ supplementation for 90 days could improve gastrointestinal barrier function, as assessed by mannitol recovery in the urine of healthy subjects and those with UC.

Regulatory oversight. This investigator-initiated interventional study was conducted under FDA approval, with JV acting as the study sponsor. The Institutional Review Board at the University of Michigan Medical School (IRBMED, HUM#00156676) provided regulatory oversight. This study was conducted as a substudy of an ongoing trial (ClinicalTrials.gov ID: NCT03869905) and under the same IRB approval; HUM#00156676 (25). The Michigan Institute for Clinical and Health Research (MICHR) supported site monitoring, data collection, and data analysis using the REDCap (Research Electronic Data Capture) platform. A dedicated Data and Safety Monitoring Committee (DSMC) conducted monthly reviews to ensure participant safety and maintenance of data integrity. The trial was registered as an interventional clinical study on ClinicalTrials.gov (NCT04855799) in April 2021, with the first participant enrolled in November 2021 and the last completing the study in March 2024. All participants provided written informed consent before enrollment and prior to any intervention. The study was conducted in accordance with established ethical standards, including the Declaration of Helsinki, CIOMS International Ethical Guidelines for Biomedical Research Involving Human Subjects, ICH Good Clinical Practice, the Belmont Report, and the U.S. Common Rule.

### Study participants

**FIGURE 1:**
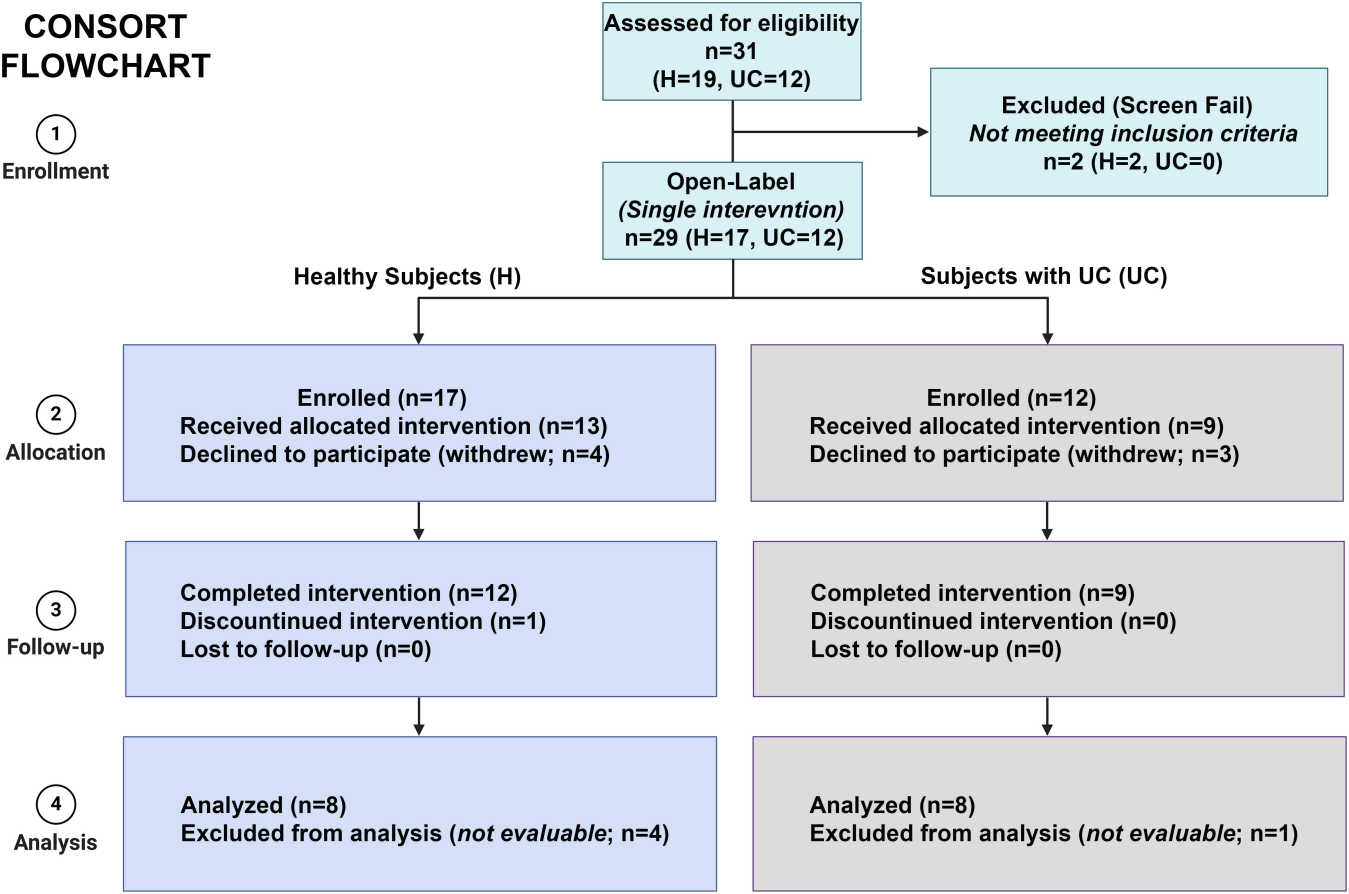
Consort flow diagram. Participant screening, enrollment, allocation, follow-up, and analysis for the 90-day open-label, single-intervention study in healthy subjects (H) and subjects with ulcerative colitis (UC). Final evaluable sample sizes are shown, along with withdrawals, discontinuations, and exclusions deemed not evaluable.

Study participants were recruited through the Michigan Medicine IBD clinics, the UMHealthResearch web portal, and by posting flyers in the hospital. Figure 1 presents a CONSORT flow diagram detailing subject enrollment, and intervention allocation. The initial intent was to include 20 evaluable subjects (10 per group/cohort) in this pilot-phase study. Although there was no prior clinical data to support this number, a previous trial with healthy subjects demonstrated that statistically significant differences in proteomic findings could be observed with as few as ten subjects per arm (24). Based on the expected dropout and screen failure rates, a total of 31 subjects were screened, and 29 subjects were enrolled in this interventional trial. Two subjects failed screening. Seven subjects withdrew, and the remaining 22 were allocated to the intervention. Of those who started on treatment, 21 completed the study, while one discontinued the treatment. Five subjects were considered unevaluable at study completion due to multiple missing urine samples, combining urine samples from two time points, or intake of fructose-containing beverages during the collection window. As a result, only sixteen subjects were fully evaluable after 90 days of intervention. This included eight subjects in each group (healthy individuals and subjects with UC) who received Aquamin^®^ for the entire 90-day period.

Participants were males or non-pregnant females in generally good health, or individuals with a confirmed diagnosis of UC. Eligible individuals with UC were either in remission or had mild disease at enrollment, as determined by the study gastroenterologist (DKT), with stable maintenance therapy and an Inflammatory Bowel Disease Questionnaire (IBDQ) score of 170 or above. The IBDQ (28) assesses health-related quality of life for UC. The IBDQ evaluates bowel, systemic, emotional, and social functions, with scores of 170 or above often indicating remission. Supplementary Table 2 presents the IBDQ scores at baseline, reflecting disease status prior to the start of the intervention.

Participants were excluded from both the healthy and UC cohorts if they were pregnant, lactating, or of childbearing potential and unwilling to use acceptable birth control throughout the study period. Individuals were also excluded if they were participating in any other interventional trial involving an investigational drug, or if they were deemed unlikely to cooperate with or comply with study procedures. Subjects with a history or diagnosis of CD, other IBDs, functional gastrointestinal disorders (including irritable bowel syndrome [IBS]), active UC within 3 months prior to enrollment (except for mild cases), gastrointestinal bleeding disorders (including those from gastric or duodenal ulcers or gastrin-secreting tumors), peptic ulcer disease with bleeding in the preceding 3 months, any gastrointestinal or colonic malignancy, kidney disease (including kidney stones or hypercalcemia), coagulopathies, hereditary hemorrhagic disorders, or neurologic disease were not eligible for participation.

Furthermore, individuals were excluded if, within 30 days prior to study entry, they had taken or were unwilling to forgo for 30 days prior to enrollment any supplements containing calcium, vitamin D (including multivitamins with these nutrients), magnesium, or fiber, non-steroidal anti-inflammatory drugs (NSAIDs; except for occasional pain management or low-dose aspirin for cardiovascular disease prevention), corticosteroids, or antibiotics.

### Study protocol

**FIGURE 2:**
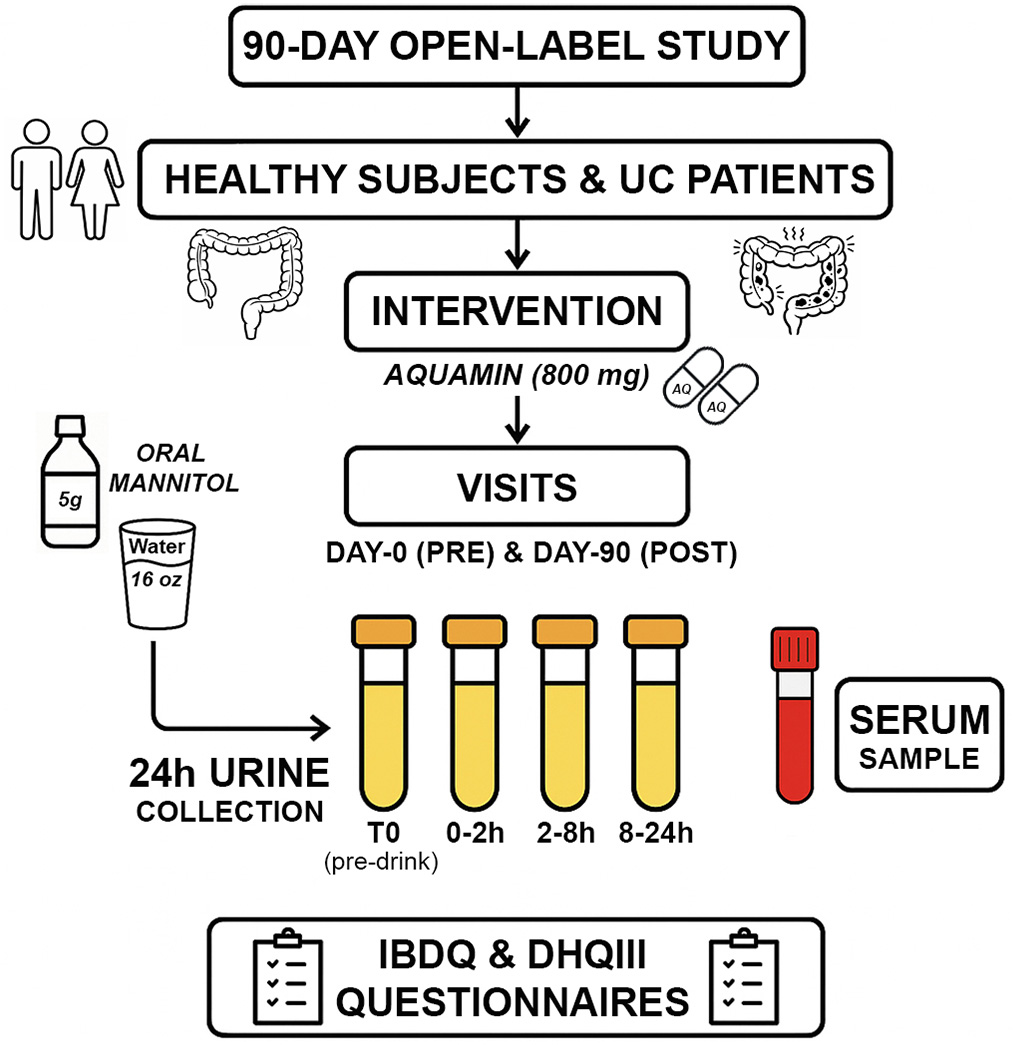
Study design and sample-collection schematic. Overview of the 90-day open-label study in healthy subjects and UC patients receiving Aquamin^®^ (800 mg of calcium). Study visits occurred at Day 0 (pre) and Day 90 (post). Gastrointestinal permeability was assessed using an oral mannitol challenge (5 g in 16 oz water) followed by 24-h urine collection partitioned into T0 (pre-drink), 0–2 h, 2–8 h, and 8–24 h fractions; a serum sample and IBDQ questionnaires were also collected. DHQIII survey was also offered to assess dietary calcium intake over the previous 12 months.

Figure 2 provides an overview of the study design. Each participant attended three study visits: (i) screening and enrollment, (ii) baseline (Day 0), and (iii) the final visit (Day 90). At the screening visit, potential participants received a full explanation of the study, after which they were administered the NIH Diet History Questionnaire III (DHQ III) (29), a comprehensive food frequency questionnaire incorporating portion size and dietary supplement intake that was used to assess baseline calcium and selected mineral intake over the previous 12 months (24, 25, 27). Participants were also queried regarding their consumption of dietary supplements, antibiotics, steroids and NSAIDs. A medical and medication history was obtained, followed by a brief physical examination. Written informed consent was obtained from eligible individuals prior to any study-related procedures. At both Visit 2 (baseline, Day 0) and Visit 3 (final, Day 90), participants provided serum samples (by venous blood draw), as well as 24-h urine samples collected. The IBDQ was completed at both visits to assess disease-related quality of life. Monthly monitoring occurred on Days 30 and 60, during which toxicity, any adverse events and adherence were assessed. Adverse events grading was based on the numerical score according to the NCI Common Terminology Criteria for Adverse Events (CTCAE), version 4.0.

#### Twenty-four-hour urine test for GI permeability assessment

Intestinal permeability was evaluated by measuring urinary recovery of D-mannitol, a non-metabolized sugar that served as the probe. The University of Michigan Research Pharmacy dispensed D-mannitol (5 g of water-soluble crystalline powder; MEDISCA Inc., product no. 0599; chemical name: 1,2,3,4,5,6-hexanehexol) and lactulose (10 g; Kristalose^®^ NDC; product no. 66220-719-01; a synthetic disaccharide from Cumberland Pharmaceuticals Inc.).

Urinary recovery of mannitol was measured at both Visit 2 (baseline, Day 0) and Visit 3 (final, Day 90) by performing a 24-h urine collection. On the morning of each visit, participants collected a pre-drink (pre-mannitol) urine sample following an overnight fast (of 6–8 h), placing the sample in a designated container kept on ice or under refrigeration. After providing the pre-drink sample, participants consumed the test solution containing 5 g D-mannitol (and 10 g lactulose) dissolved in 16 oz of water, ingested over a period of 15–20 min, immediately before the start of the 24-h urine collection. They were allowed to take one additional cup (16 oz) of water before or during that period. Following administration of the test solution, participants abstained from both food and beverage intake (other than water) for the first 8 h of the subsequent 24-h urine collection period. Participants were allowed to drink two additional cups of water (16 oz each) after the first 2 h (during the next 6 h) of urine collection. All urine excreted during the 24-h period was collected in separate containers according to the following schedule: pre-mannitol urine in container #1, the first 2 h in container #2, h 2–8 in container #3, and hours 8–24 in container #4. All containers were kept chilled throughout the collection period. At the end of the initial 8-h window, participants resumed their regular food and water intake, avoiding only fructose-containing foods and documenting all fluid consumption to account for variability.

Participants were instructed to maintain their usual diet and fluid intake the day prior to testing, with the exception of strict avoidance of specific foods and beverages for at least 24 h before the test. These restricted items included fruits, fruit juices, jams, jellies, foods and beverages sweetened with high-fructose corn syrup, artificial sweeteners, alcoholic drinks, dairy products, dietetic chocolate, honey, mushrooms, legumes (including beans and peanuts), celery, and chewing gum. Additionally, all marijuana-related products and mannitol-containing foods or supplements were prohibited for at least 48 h prior to and during testing.

Upon returning to the study center, participants submitted their complete 24-h urine collections. The total volume of each sample was measured, and aliquots were prepared for storage. All samples were promptly frozen at –80°C. Mannitol recovery and intestinal permeability were assessed at the end of the study by quantifying urinary mannitol concentration using the ELISA kit (ChromaDazzle D-Mannitol Assay Kit by AssayGenie; Cat# BA0133).

### Serum biomarkers

A comprehensive metabolic panel was performed to assess serum biomarkers, including total albumin, bilirubin, aspartate aminotransferase (AST), alanine aminotransferase (ALT), and ALP, along with additional analytes relevant to liver and kidney function. For participants with UC, CRP levels were measured concurrently with the metabolic panel. All serum analyses were conducted by the Michigan Medicine laboratory, which generated individual reports for each participant in accordance with established standard operating procedures. The comprehensive metabolic panel was evaluated primarily to monitor safety, although CRP and ALP measurements were also clinically relevant for routine UC management (30, 31).

### Statistical evaluation

This pilot study was designed to assess feasibility and tolerability of Aquamin^®^, as well as to evaluate biomarkers related to gastrointestinal permeability. We anticipated that the study would reveal trends useful for estimating effect sizes and guiding the formulation of research hypotheses for subsequent, fully powered, large-scale trials. It was hypothesized that Aquamin^®^ would exert a positive effect on mannitol recovery, with the magnitude of the effect varying among participants.

Based on the study design, pre- and post-intervention values for various serum analytes included in the metabolic panel, as well as CRP and urinary mannitol levels (measured by ELISA), were obtained from each subject. Group means and standard deviations were calculated for each endpoint, and within each cohort, pre- versus post-intervention data were analyzed using paired *t*-tests (at the 95% confidence level). Subsequently, an independent-samples *t*-test was conducted to compare the pre-post change score between the two cohorts. To account for inflated Type I error due to testing multiple endpoints, Tukey’s multiple comparisons test was used to determine significance. A *p*-value < 0.05 was considered significant. Statistical analyses were conducted using GraphPad Prism v10.2. Given the small sample size, analyses were not adjusted for baseline sociodemographic variables, including gender, or for initial dietary calcium intake levels.

## Results

### Participant demographics and baseline characteristics

Out of 31 screened subjects, sixteen subjects (eight healthy individuals and eight with UC, either in remission or with mild disease) completed the study and were considered evaluable. Supplementary Table 3 (and Figure 1) presents demographic profiles (including ethnicity) of subjects who were assessed for eligibility and enrolled in the 90-day interventional study. Of these 31 subjects, sixteen were female and fifteen were male, with comparable numbers of males and females enrolled in both the healthy and UC cohorts. The mean age of healthy subjects was 31.7 (± 12.0) years, while the mean age for those with UC was 41.9 (± 12.7) years at the start of the study. Body mass index (BMI) remained unchanged in both cohorts over the 90-day study period compared to baseline.

Supplementary Table 4 presents average daily intakes for eight minerals, estimated from participant dietary reports over the previous 12 months using the NIH DHQ III, compared to recommended guidelines. Both healthy subjects and those with UC exhibited similar intake patterns for most minerals, generally meeting or exceeding suggested targets for calcium, copper, zinc, iron, and selenium, but falling short of recommendations for potassium and vitamin D. Magnesium intake was below recommended levels in healthy subjects but adequate among UC subjects. Notably, while vitamin D intake from food and supplements (estimated by DHQIII) remained substantially below recommended levels in both groups, the DHQIII assessment did not include questions related to sun exposure (see Supplementary Table 4).

### Participant safety

For safety assessment, subjects reported side effects experienced during the study, and serum samples were collected to evaluate liver and kidney function.

Adverse events (AEs) were reported in six healthy subjects (out of 13) and six UC subjects (out of 9), with a total of 20 and 10 events, respectively (Supplementary Table 5). No serious AEs occurred during the study. Most events were considered unrelated to the study except for gastrointestinal symptoms which included flatulence, abdominal discomfort, and constipation. These events were mild and were classified as CTCAE Grade 1. These results align with our previous findings in both healthy individuals (24, 27) and subjects with UC (25), indicating that Aquamin^®^ is safe for use and unlikely to pose safety or tolerability concerns. Three adverse events were also reported following mannitol and lactulose administration, including diarrhea (loose stool) in one UC subject (Supplementary Table 5).

**FIGURE 3:**
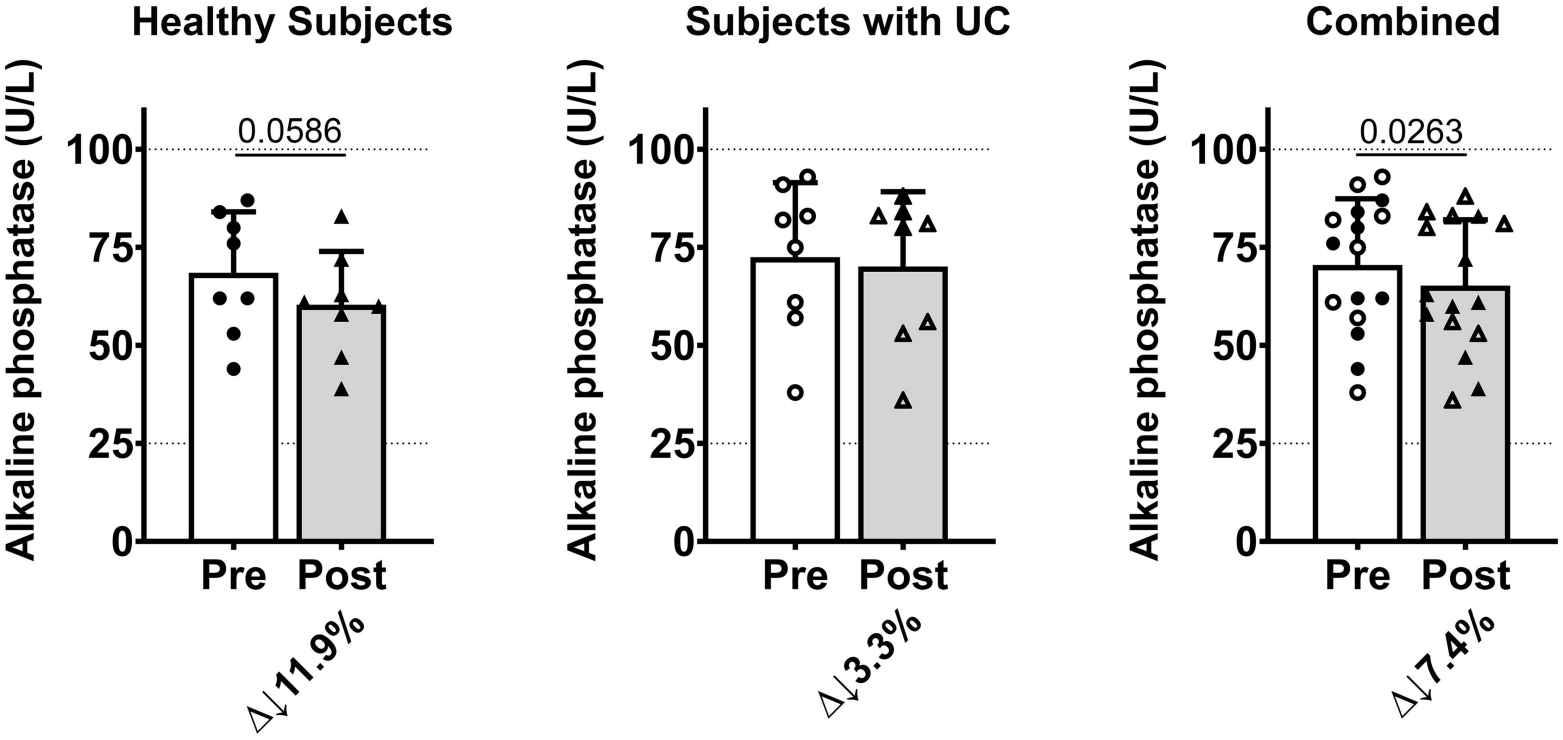
Serum alkaline phosphatase pre- vs. post-intervention. Serum alkaline phosphatase (ALP; U/L) was measured at baseline (Pre) and after 90 days (Post) in healthy subjects, UC subjects, and the combined analysis. Bars show the group mean with standard deviation error bars, and points indicate individual participant values. Pre–post percent change (Δ) is shown below each panel, and two-tailed paired *t*-test *P* values for the pre–post comparison are shown above.

A metabolic panel was performed on serum samples from each participant at study initiation and after the final dose of Aquamin^®^ on Day 90. Results are presented in Table 1. Serum metabolic panel values for both healthy participants and those with UC remained within normal reference ranges from baseline to post-intervention, with no clinically significant changes observed over the 90-day study period. Mean values for major safety markers—including liver enzymes (AST, ALT, ALP), kidney function indicators (BUN and creatinine), and electrolytes—were stable, suggesting no adverse impact of the intervention. Overall, no trends indicating treatment-related hepatic or renal toxicity were observed in either cohort, consistent with findings from our previous trials (24, 25, 27). Also, in line with what was reported previously (25), there was a small but consistent decrease in serum ALP levels with Aquamin^®^ over the course of the 90-day treatment period: 11.9% in healthy subjects and 3.3% in UC subjects. When pre- and post-intervention differences in serum ALP from all 16 participants (all on Aquamin^®^) were analyzed together using a paired *t*-test, the overall decrease (7.4%) reached statistical significance (*p* = 0.026; Figure 3).

**TABLE 1:** Serum chemistry (metabolic panel).

Analyte	Reference range	Healthy subjects		Ulcerative colitis	
		Pre	Post	Pre	Post
Albumin	(3.5–5.0 g/dL)	4.51 ± 0.31	4.44 ± 0.18	4.33 ± 0.35	4.48 ± 0.35
Total protein	(6.0–8.3 g/dL)	6.69 ± 1.10	6.79 ± 0.54	7.09 ± 0.48	7.13 ± 0.51
AST	(M:14–20 F:10–36 U/L)	22.88 ± 3.56	24.00 ± 9.10	24.50 ± 9.65	22.38 ± 8.18
ALT	(M:10–40 F:7–35 U/L)	23.38 ± 11.06	26.63 ± 17.06	26.63 ± 26.21	20.75 ± 11.35
ALP	(25–100 U/L)	68.50 ± 15.57	60.38 ± 13.61	72.50 ± 19.02	70.13 ± 19.09
Bilirubin	(0.0–1.4 mg/dL)	1.13 ± 0.70	1.06 ± 0.89	0.70 ± 0.32	0.83 ± 0.35
Sodium	(136–145 mmol/L)	139.88 ± 1.13	140.88 ± 1.25	140.75 ± 1.28	139.00 ± 2.14
Potassium	(3.5–5.2 mmol/L)	4.34 ± 0.35	4.40 ± 0.33	4.16 ± 0.24	4.23 ± 0.15
Chloride	(96–106 mmol/L)	105.50 ± 1.69	107.13 ± 1.81	107.38 ± 2.20	106.50 ± 1.69
Carbon dioxide	(23–30 mmol/L)	28.13 ± 1.13	27.88 ± 2.10	27.00 ± 2.20	27.00 ± 1.93
BUN	(10–20 mg/dL)	11.75 ± 3.28	12.75 ± 2.31	13.13 ± 2.80	13.00 ± 2.83
Creatinine	(M:0.6–1.2 F:0.5–1.1 mg/dL)	0.94 ± 0.16	0.93 ± 0.13	0.80 ± 0.15	0.93 ± 0.14
BUN-creatinine ratio	(10:1–20:1)	11.30 ± 3.83	12.90 ± 3.18	16.82 ± 4.96	14.00 ± 2.19
Glucose	(<100 mg/dL)	91.13 ± 12.97	88.00 ± 7.35	85.13 ± 12.36	89.88 ± 7.55
Calcium	(8.8–10.4 mg/dL)	9.75 ± 0.35	9.69 ± 0.29	9.46 ± 0.33	9.49 ± 0.37

A comprehensive serum metabolic panel was performed for each participant at baseline (pre) and Day 90 (post) at the Michigan Medicine Pathology and Clinical Laboratory as part of the safety assessment. The normal (reference) ranges for each analyte are listed at the top of the panel. For each analyte, values are reported as the mean and standard deviation, calculated from the measurements of all subjects at each time point. For AST, ALT, and creatinine, reference ranges differ for males and females; however, due to the small sample size (*n* = 8 in each group), data from both sexes were combined to report a single mean value for each analyte. AST, Aspartate aminotransferase; ALT, Alanine aminotransferase; ALP, Alkaline phosphatase; BUN, Blood Urea Nitrogen.

Serum CRP was assessed only in subjects with UC (in remission or with mild disease). At study completion, CRP values were 0.39 ± 0.13, compared to baseline values of 0.40 ± 0.19, representing a 3% decrease. This reduction is similar to that observed in our previous study of UC patients (in remission or with mild disease) after 90 days of Aquamin^®^ intervention (25). Supplementary Table 2 presents the IBDQ scores, before and after 90 days of intervention. Average group scores changed little between Day 0 and Day 90. Among UC participants who received Aquamin^®^ for 90 days, mean scores on the full questionnaire increased from 191.6 ± 15.3 to 194.3 ± 20.5, a modest trend consistent with our prior study involving UC subjects in remission or with mild disease (25).

### Gastrointestinal permeability assessment

**FIGURE 4:**
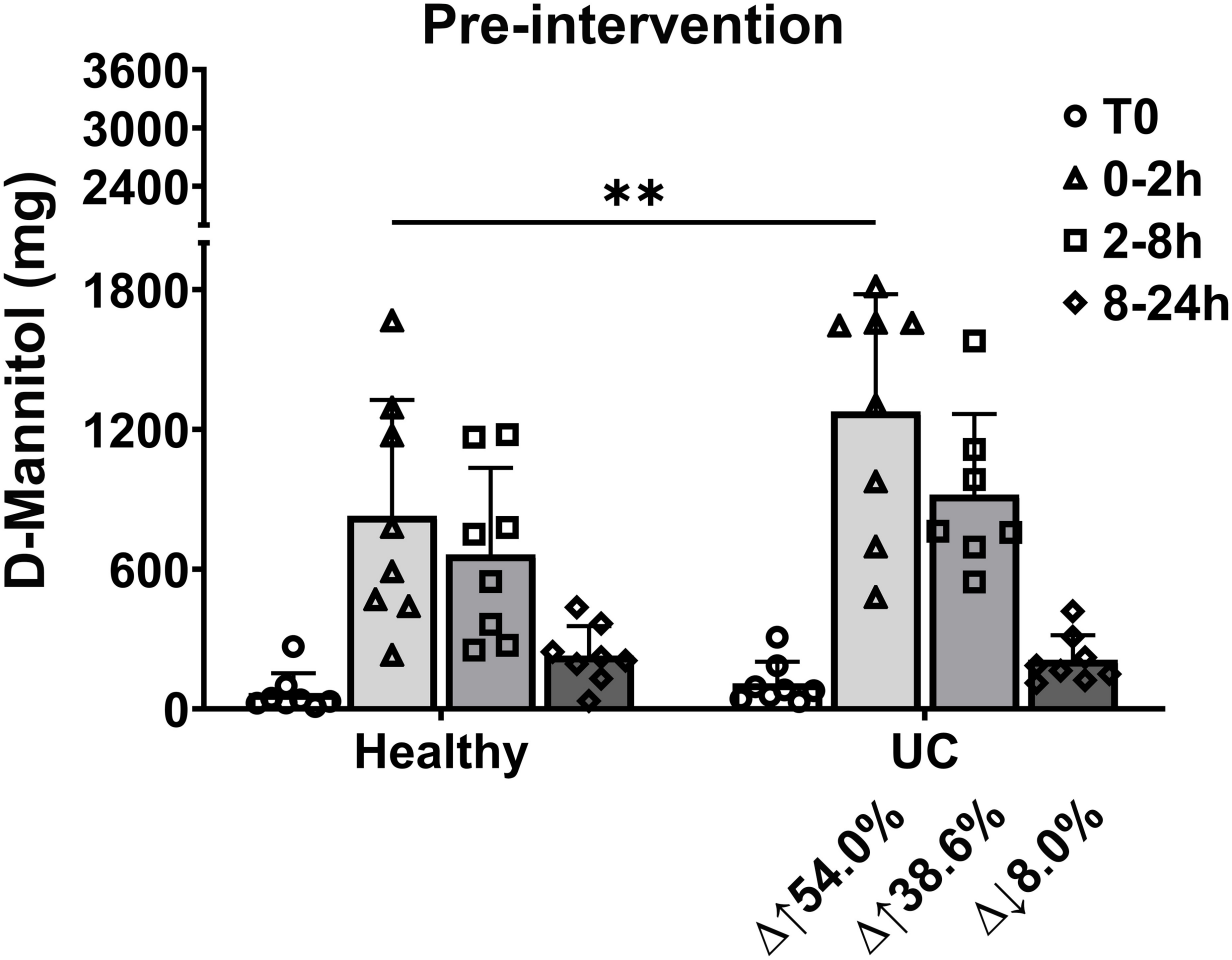
Baseline urinary D-mannitol excretion in healthy subjects and UC patients. Pre-intervention urinary D-mannitol (mg) recovered in sequential urine collections (T0, 0–2 h, 2–8 h, 8–24 h) following an oral mannitol challenge is shown for healthy subjects versus UC subjects. Bars show the group mean with standard deviation error bars; symbols denote individual participant values. Percent difference (Δ) for UC relative to the corresponding healthy interval is shown below the UC bars. ** indicates significance with *P* < 0.01 for the UC vs. healthy mannitol excretion comparison at 0–2 h.

**FIGURE 5:**
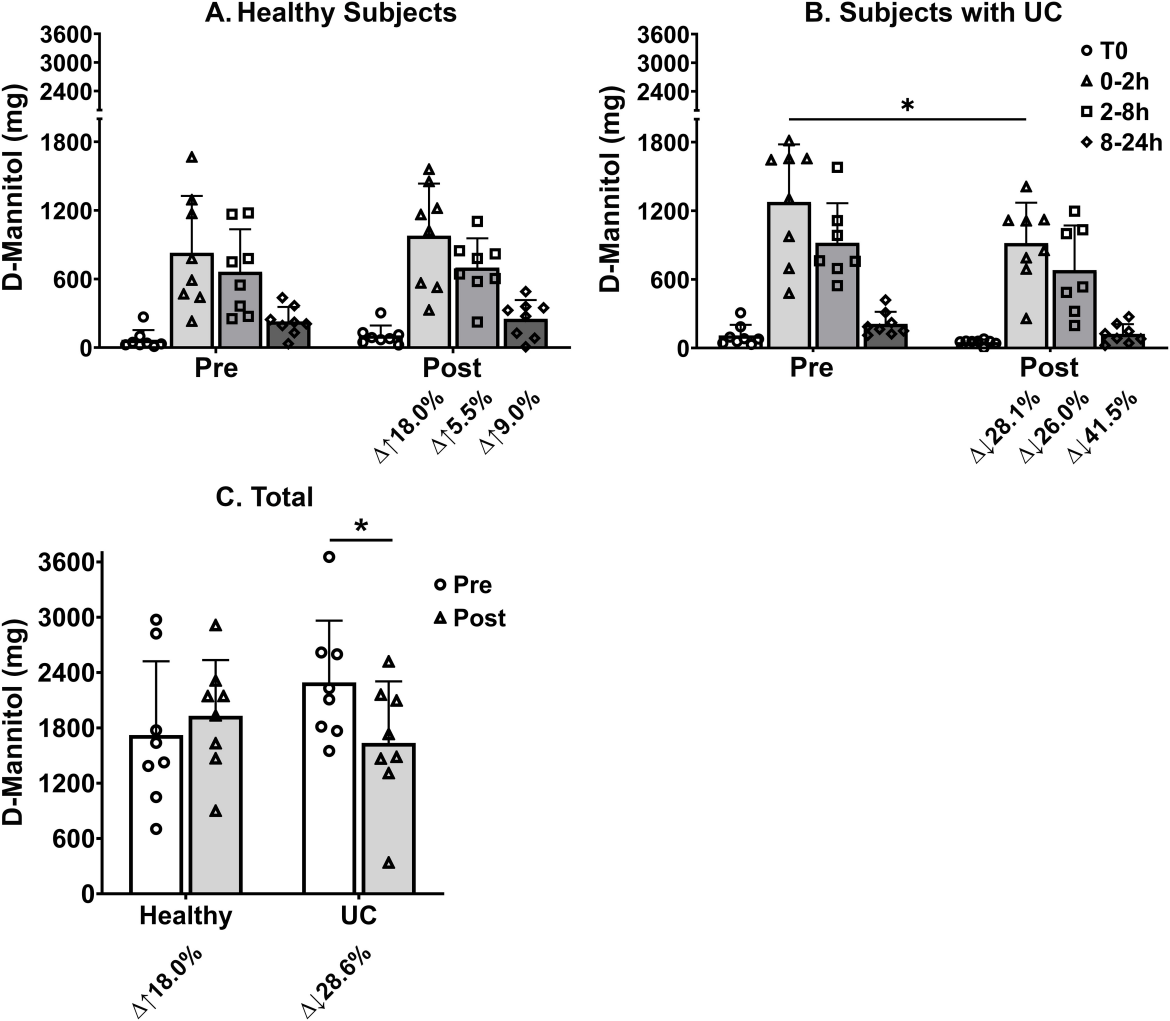
Effect of Aquamin^®^ intervention on urinary D-mannitol excretion. **(A)** Healthy subjects and **(B)** UC subjects: urinary D-mannitol (mg) in each collection interval (T0, 0–2 h, 2–8 h, and 8–24 h) at baseline (Pre) and after 90 days (Post). **(C)** Total 24-h D-mannitol excretion (mg) pre- and post-intervention for each group. Bars show group mean with standard deviation error bars; symbols indicate individual participant values. Pre-post percent change (Δ) from baseline is shown; * indicates significance with *P* < 0.05 for the indicated pre–post comparisons (in **B:** for UC subjects, pre-post difference is significant at 0–2 h and in **C:** for UC subjects, total 24-h pre-post difference is significant).

To assess *in vivo* gastrointestinal permeability, we administered an oral mannitol dose (5 g) and measured urinary mannitol levels via ELISA at multiple time points. Data are presented in Figures 4, 5.

Figure 4 compares pre-intervention (prior to Aquamin^®^ treatment) urinary mannitol excretion in healthy controls and subjects with UC across defined intervals—i.e., at baseline (prior to mannitol consumption) and at 0–2 h, 2–8 h and 8–24 h post-mannitol consumption. Baseline (T0; pre-mannitol drink) levels were minimal in both groups. In healthy participants, mannitol recovery peaked in the 0–2-h interval (mean 829 mg), was moderate at 2–8 h and declined further by 8–24 h. UC subjects demonstrated significantly greater mannitol excretion at 0–2 h (mean 1277 mg, *p* = 0.0064 compared to healthy counterparts at 0–2 h), indicative of increased early intestinal permeability. Elevated excretion persisted, though at reduced levels, in subsequent intervals. Percent change analysis in UC subjects showed a 54.0% increase at 0–2 h and a 39% increase at 2–8 h compared to healthy subjects at the same time points. These results reveal a more substantial and more rapid urinary mannitol recovery in the UC cohort, most evident in the first 2 h post-administration.

Figure 5 compares urinary mannitol excretion in healthy subjects and those with UC before and after 90 days of intervention with Aquamin^®^. In healthy subjects (Figure 5A), no significant differences in urinary mannitol excretion were observed between pre- and post-intervention across all collection intervals. Post-intervention mannitol levels were slightly increased. In UC subjects (Figure 5B), post-intervention mannitol excretion during the 0–2-h interval was significantly reduced compared to pre-intervention values (*p* = 0.015), with an overall percent decrease of 28%. Mean urinary mannitol levels decreased from 1277 mg at pre-intervention to 917 mg at post-intervention. Mannitol levels continued to decline in post-intervention samples across the next two collection time points. Figure 5C provides a summary of the total urinary mannitol values in pre- and post-treatment specimens from the two participant groups. The combined analysis (Figure 5C) confirms a significant reduction (*p* = 0.0236) in total urinary mannitol recovery post-intervention for UC. That is, an overall reduction of 29% (from approximately 2300 mg at baseline to 1637 mg on Day 90) was observed in the UC participant group. In healthy subjects, total mannitol recovery was virtually unchanged at Day 90 as compared to pre-Aquamin^®^ intervention.

Supplementary Figures 1, 2 present individual participant data as spaghetti plots to facilitate individual-level comparison. Supplementary Figures 1A,B show pre-intervention data for healthy controls and subjects with UC, whereas Supplementary Figures 1C,D show post-intervention data from the same subjects. At baseline, prior to starting the intervention, mannitol levels were higher in subjects with UC than in healthy controls, particularly at the 0–2-h time point (Figure 4 and Supplementary Figure 1B). Post-intervention, mannitol levels were similar between the two groups (Supplementary Figures 1C,D). Supplementary Figures 2A–D present pre- and post-intervention spaghetti plots for both groups and show a clear reduction in mannitol levels in post-intervention samples from subjects with UC (Supplementary Figures 2C,D), which is also shown in Figure 5B.

## Discussion

A breakdown in the permeability barrier of the gastrointestinal tract is a feature of UC (1–3). While bowel inflammation is recognized as a cause of barrier dysfunction, increased permeability has been observed in some UC patients even in the absence of overt inflammation (32, 33). Increased permeability has also been shown to predict disease recurrence among IBD patients in remission (34) and has been correlated with subsequent disease development in first-degree relatives of IBD sufferers (35). Thus, barrier breakdown may promote inflammation in addition to being its result. Regardless of whether barrier dysfunction triggers inflammation or is a consequence of it, improving barrier integrity is likely to be beneficial. Numerous dietary supplements have been purported to reduce “leaky gut” (2, 9), but to date, none has proven sufficiently effective to warrant widespread recommended use. Here we show that daily consumption of a multi-mineral product over a 90-day period retarded mannitol transport across the gastrointestinal wall in a small cohort of UC patients (in remission or with mild UC). Results from our previous organoid culture studies (18–23) and findings from two interventional trials (24, 25) demonstrated upregulation of multiple proteins involved in colonic barrier integrity. Taken together with these previous data, the current findings allow us to suggest improved barrier function with Aquamin^®^ use.

Building on these considerations, our evaluation of gut permeability in participants with mild UC or those in remission, compared with healthy subjects, demonstrates a characteristic difference at baseline. Participants with UC had greater early uptake and transfer of mannitol from the gut into the bloodstream (reflected by higher 0–2-h urinary recovery), consistent with increased intestinal permeability (a “leakier” barrier) compared to healthy controls (Figure 4). In participants with UC, the post-intervention decreases in the 0–2-h and total (24-h) mannitol recovery (Figure 5) indicate that Aquamin^®^ intervention was associated with reduced permeability and improved barrier function. In contrast, the lack of change in healthy subjects suggests no material effect when baseline permeability is already normal.

Gastrointestinal permeability refers to the transport of substances across the gut wall through both transcellular and paracellular mechanisms (36). Mammalian cells lack specific cell surface transporters for mannitol, but passive diffusion across the cellular layer of the gut still occurs (37–39). Due to its small size and inert nature, mannitol can also be transported paracellularly through intact tight junctional pores in healthy tissue (40). No doubt, mannitol passage can also occur non-specifically when there is overt damage to the intestinal wall. Measuring urinary mannitol excretion over a 24-h period following ingestion of the probe (as done here) does not allow us to distinguish among these possibilities. Thus, the current study does not elucidate how Aquamin^®^ affects the permeability barrier in the gut.

To gain insight into how a mineral supplement might function to improve permeability control, our previous organoid culture studies are instructive. In organoid culture, inclusion of Aquamin^®^ in the medium had only a modest effect on tight junctional protein expression, but strongly upregulated adherens junction and desmosomal cadherins as well as other proteins necessary for cell-cell adhesion (18–23). Basement membrane components and proteins that mediate cell-matrix adhesion via hemidesmosomes and focal adhesions were also upregulated (18, 19, 21). Finally, proteins that make up the mucinous layer were enhanced in the presence of Aquamin^®^ (18, 19, 22, 23). Not surprisingly, given this wide range of affected proteins, an increase in TEER as well as an increase in tissue cohesion was seen in Aquamin^®^-treated organoids (20–22). Of interest, the response to Aquamin^®^ was as robust in organoids derived from healthy tissue as it was in UC-derived organoids (18, 19). These data suggest that, in organoid culture at least, minerals in the algae-derived product act on basic physiological processes that drive barrier protein generation. Enhanced barrier protein expression likely makes the tissue more resistant to inflammatory injury initially and allows the tissue to regain normal barrier function more rapidly when injury does occur. A role for Aquamin^®^ that is independent of a direct effect on inflammation is, therefore, suggested. Whether these *in vitro* changes in protein expression have impact on the findings presented here with regard to mannitol transport is yet to be fully substantiated. All we know for sure is that changes in barrier protein expression seen in organoid culture were also seen in biopsies taken from individuals after daily Aquamin^®^ ingestion for 90 or 180 days (24, 25).

In the same organoid culture studies, a proteomic platform was used to assess a wide range of protein changes in response to Aquamin^®^ treatment. Several proteins with anti-inflammatory potential were induced while other proteins that are known to promote inflammation were downregulated (18, 19, 22, 23). Thus, while we hypothesize that the major effect of Aquamin^®^ is on barrier protein expression, a direct anti-inflammatory role is also possible. These two effects are not mutually exclusive. Either could lead to improved permeability control.

In addition to protein expression changes, Aquamin^®^ ingestion was also shown to alter the microbial profile in the colon and reduce the level of several toxic bile acids (27). Both effects could contribute to improved intestinal permeability and gut health. Thus, caution should be taken in interpreting Aquamin^®^’s primary effect as being simply due to increased barrier protein expression or a direct effect on inflammation. Ultimately, it is not unreasonable to suggest that a multi-mineral product could affect gastrointestinal health through multiple independent or overlapping mechanisms.

As part of the current study, as well as in our two previous trials (24, 25), all subjects had a serum metabolic panel performed at the start and end of the interventional period. In none of the three trials were detectable changes observed in any of the metabolites that would indicate a safety concern. Of more interest, in both the recent trial with UC patients (25) and in the current study, serum ALP levels decreased over the course of treatment with Aquamin^®^. In the current study (following a 90-day intervention), the decline was statistically significant (*p* < 0.05). In the recent trial with UC patients (25), the decline in serum ALP was seen after both 90 days and 180 days of intervention but it reached a level of statistical significance only after 180 days. In that study, a second assay measuring the intestine-specific isoform of ALP (ALPI) in serum demonstrated a comparable decline over the 180-day treatment period. In parallel with the decline in serum ALPI levels, colonic tissue ALPI levels were higher after 180 days of Aquamin^®^ treatment period than in pretreatment samples (25). This was not observed in UC patients receiving placebo. The significance of this finding is not fully understood for a number of reasons. Most importantly, while ALP could modulate colon inflammation in a number of ways (41–44), the relationship between elevated ALP and inflammatory bowel disease (including CD as well as UC) is simply not fully understood. Likewise, how elevated ALP affects primary sclerosing cholangitis (PSC), which is often seen in both UC and CD (45, 46), is not understood.

While the current study suggests that Aquamin^®^ can improve at least one parameter of barrier function *in vivo*, the study has limitations. First, the small sample size limited statistical power to detect changes while the high dropout rate could, in theory, have introduced bias. It should be noted that subject “drop-out” had more to do with the urine collection procedure than with taking daily dose of intervention. Finally, the open-label nature of the study deprived us from having a control group against which to compare the magnitude of the supplement-induced changes.

A second limitation—the use of mannitol alone as a probe for permeability status—may identify alterations in permeability but does not address mechanism of action. Importantly, it is not known if large moieties such as intact microbial cells, cellular components or food-derived allergens would show the same reduced paracellular passage in response to Aquamin^®^ as seen here with the monosaccharide. Including larger sugar molecules such as lactulose could be beneficial in this regard as some studies have demonstrated that the lactulose/mannitol ratio provides data not obtainable with mannitol alone (1–3). In fact, we expended significant effort in this regard, but could not obtain reliable lactulose data in urine samples with existing ELISA kits to calculate lactulose:mannitol ratio. While the use of mannitol alone has limitations, the consistency of the findings presented here, and the results from past studies with this probe (47–50) clearly attest to its utility.

These limitations, while important, point out the difficulties in carrying out a pilot-phase clinical investigation. The overall length of time it took to complete the study and the overall cost made a larger trial impossible. With the data obtained here, a more definitive study should be possible. The fact that we have observed no study agent-related serious adverse events, no adverse changes in the metabolic panel readouts and no tolerability issues either here or in two previous trials (25, 27) supports moving forward with a larger trial and longer interventional period. Increasing the amount of Aquamin^®^ provided could enhance efficacy and utilization of a placebo-control group would add to the value of study findings. As a way to begin addressing these limitations, we have initiated a new trial (ClinicalTrials.gov: NCT07501078) in UC patients with a J-pouch (following ileal pouch-anal anastomosis) to study effect of Aquamin^®^ on the prevention of recurrence of inflammation of the intestinal pouch lining, a common downstream complication of this procedure.

In summary, our previous *ex vivo* and *in vivo* studies have demonstrated increased barrier protein expression in the gastrointestinal tract with Aquamin^®^ (18–25). Here we present evidence for barrier improvement *in vivo*. While these investigations were conducted primarily to support the development of Aquamin^®^ as an ancillary treatment for UC, barrier dysfunction is recognized as a pathophysiological component of many other gastrointestinal disorders. In addition to UC and CD, these include malabsorptive conditions such as celiac disease (51, 52), IBS (53), and diseases affecting the liver and pancreas (54–56). Importantly, increased intestinal permeability has also been observed in various neurological disorders involving gut-brain axis—including Alzheimer’s disease (57), Parkinson’s disease (58), amyotrophic lateral sclerosis (59), and anxiety/depression (60). Finally, recent studies have suggested that gastrointestinal barrier disruption can serve as a predictor of incipient transplant rejection in allogeneic tissue transplantation (61). Given Aquamin^®^’s demonstrated safety and tolerability profile and its potential to enhance barrier integrity, it may offer therapeutic benefit as an adjuvant and barrier-targeted intervention in any of these disorders where increased intestinal permeability is implicated in disease activity or progression. Future studies to expand the limited scope of the present trial and to explore its application beyond UC are warranted.

## Data Availability

The original contributions presented in this study are included in this article/Supplementary material, further inquiries can be directed to the corresponding author.

## Supplementary material

Supplementary Figure 1 **(A,B)** Pre-intervention (Day 0). **(C,D)** Post-intervention (Day 90) urinary D-mannitol excretion in healthy subjects and patients with UC. Pre- and post-intervention urinary D-mannitol (mg) recovered in sequential urine collections (T0, 0–2 h, 2–8 h, and 8–24 h) following an oral mannitol challenge are shown for healthy subjects versus subjects with UC in spaghetti plots. **P* < 0.05 for the UC vs healthy comparison of mannitol excretion at 0–2 h prior to intervention.

Supplementary Figure 2 Effect of Aquamin^®^ intervention on urinary D-mannitol excretion in healthy subjects **(A,B)** and UC subjects **(C,D)** at baseline (Day 0; Pre-intervention) and Day 90 (Post-intervention). Urinary D-mannitol (mg) in each collection interval (T0, 0–2 h, 2–8 h, and 8–24 h) is shown at baseline (Pre-intervention) and after 90 days (Post-intervention) in spaghetti plots. * indicates *P* < 0.05 for the indicated pre–post comparisons in UC subjects; the pre-post difference was significant at 0–2 h.

## References

[1] Dunleavy KA Rypstra CR Busciglio I Eckert D Ryks M Omerdic E et al. Intestinal permeability in vivo in patients with inflammatory bowel disease: comparison of active disease and remission. *Inflamm Bowel Dis*. (2025) 31:2511–20. 10.1093/ibd/izaf043 40067071 PMC12455586

[2] Camilleri M. Leaky gut: mechanisms, measurement and clinical implications in humans. *Gut*. (2019) 68:1516–26. 10.1136/gutjnl-2019-318427 31076401 PMC6790068

[3] Rao AS Camilleri M Eckert DJ Busciglio I Burton DD Ryks M et al. Urine sugars for in vivo gut permeability: validation and comparisons in irritable bowel syndrome-diarrhea and controls. *Am J Physiol Gastrointest Liver Physiol.* (2011) 301:G919–28. 10.1152/ajpgi.00168.2011 21836056 PMC3220318

[4] Vindigni SM Zisman TL Suskind DL Damman CJ. The intestinal microbiome, barrier function, and immune system in inflammatory bowel disease: a tripartite pathophysiological circuit with implications for new therapeutic directions. *Ther Adv Gastroenterol*. (2016) 9:606–25. 10.1177/1756283X16644242 27366227 PMC4913337

[5] Chelakkot C Ghim J Ryu SH. Mechanisms regulating intestinal barrier integrity and its pathological implications. *Exp Mol Med*. (2018) 50:1–9. 10.1038/s12276-018-0126-x 30115904 PMC6095905

[6] Ahmad R Sorrell MF Batra SK Dhawan P Singh AB. Gut permeability and mucosal inflammation: bad, good or context dependent. *Mucosal Immunol.* (2017) 10:307–17. 10.1038/mi.2016.128 28120842 PMC6171348

[7] Lee JY Wasinger VC Yau YY Chuang E Yajnik V Leong RW. Molecular pathophysiology of epithelial barrier dysfunction in inflammatory bowel diseases. *Proteomes*. (2018) 6:17. 10.3390/proteomes6020017 29614738 PMC6027334

[8] Salim SY Söderholm JD. Importance of disrupted intestinal barrier in inflammatory bowel diseases. *Inflamm Bowel Dis*. (2011) 17:362–81. 10.1002/ibd.21403 20725949

[9] Matar A Abdelnaem N Camilleri M. Bone broth benefits: How its nutrients fortify gut barrier in health and disease. *Dig Dis Sci*. (2025) 70:1951–61. 10.1007/s10620-025-08997-x 40180691

[10] Aslam MN Paruchuri T Bhagavathula N Varani J. A mineral-rich red algae extract inhibits polyp formation and inflammation in the gastrointestinal tract of mice on a high-fat diet. *Integr Cancer Ther*. (2010) 9:93–9. 10.1177/1534735409360360 20150219 PMC2861409

[11] Aslam MN Bergin I Naik M Paruchuri T Hampton A Rehman M et al. A multimineral natural product from red marine algae reduces colon polyp formation in C57BL/6 mice. *Nutr Cancer*. (2012) 64:1020–8. 10.1080/01635581.2012.713160 23035966 PMC3660990

[12] Aslam MN Bergin I Naik M Hampton A Allen R Kunkel SL et al. A multi-mineral natural product inhibits liver tumor formation in C57BL/6 mice. *Biol Trace Elem Res*. (2012) 147:267–74. 10.1007/s12011-011-9316-2 22222483 PMC3360994

[13] Varani J McClintock SD Knibbs RN Harber I Zeidan D Jawad-Makki MAH et al. Liver protein expression in NASH mice on a high-fat diet: response to multi-mineral intervention. *Front Nutr*. (2022) 9:859292. 10.3389/fnut.2022.859292 35634402 PMC9130755

[14] Hampton AL Aslam MN Naik MK Bergin IL Allen RM Craig RA et al. Ulcerative dermatitis in C57BL/6NCrl mice on a low-fat or high-fat diet with or without a mineralized red-algae supplement. *J Am Assoc Lab Anim Sci.* (2015) 54:487–96. 26424246 PMC4587616

[15] Aslam MN Varani J. The western-style diet, calcium deficiency and chronic disease. *J Nutr Food Sci.* (2016) 6:3. 10.4172/2155-9600.1000496

[16] Nadeem Aslam M Bassis CM Zhang L Zaidi S Varani J Bergin IL. Calcium reduces liver injury in mice on a high-fat diet: alterations in microbial and bile acid profiles. *PLoS One*. (2016) 11:e0166178. 10.1371/journal.pone.0166178 27851786 PMC5113033

[17] Aviello G Amu S Saunders SP Fallon PG. A mineral extract from red algae ameliorates chronic spontaneous colitis in IL-10 deficient mice in a mouse strain dependent manner. *Phytother Res*. (2014) 28:300–4. 10.1002/ptr.4989 23554071

[18] Attili D McClintock SD Rizvi AH Pandya S Rehman H Nadeem DM et al. Calcium-induced differentiation in normal human colonoid cultures: cell-cell / cell-matrix adhesion, barrier formation and tissue integrity. *PLoS One*. (2019) 14:e0215122. 10.1371/journal.pone.0215122 30995271 PMC6469792

[19] Aslam MN McClintock SD Attili D Pandya S Rehman H Nadeem DM et al. Ulcerative colitis-derived colonoid culture: a multi-mineral-approach to improve barrier protein expression. *Front Cell Dev Biol*. (2020) 8:577221. 10.3389/fcell.2020.577221 33330453 PMC7719760

[20] McClintock SD Attili D Dame MK Richter A Silvestri SS Berner MM et al. Differentiation of human colon tissue in culture: effects of calcium on trans-epithelial electrical resistance and tissue cohesive properties. *PLoS One*. (2020) 15:e0222058. 10.1371/journal.pone.0222058 32134920 PMC7058309

[21] Varani J McClintock SD Aslam MN. Cell-matrix interactions contribute to barrier function in human colon organoids. *Front Med*. (2022) 9:838975. 10.3389/fmed.2022.838975 35360746 PMC8960989

[22] Varani J McClintock SD Nadeem DM Harber I Zeidan D Aslam MN. A multi-mineral intervention to counter pro-inflammatory activity and to improve the barrier in human colon organoids. *Front Cell Dev Biol*. (2023) 11:1132905. 10.3389/fcell.2023.1132905 37476158 PMC10354648

[23] Aslam MN McClintock SD Moraga G Nadeem DM Harber I Varani J. Proteomic profile of human colon organoids: effects of a multi-mineral intervention alone and in the presence of pro-inflammatory and anti-inflammatory treatments. *Front Gastroenterol*. (2025) 4:1592669. 10.3389/fgstr.2025.1592669 41822286 PMC12952359

[24] Aslam MN McClintock SD Jawad-Makki MAH Knuver K Ahmad HM Basrur V et al. A multi-mineral intervention to modulate colonic mucosal protein profile: results from a 90-day trial in human subjects. *Nutrients*. (2021) 13:939. 10.3390/nu13030939 33799486 PMC8002192

[25] Aslam MN Turgeon DK Appelman HD Stidham R McClintock S Allen R et al. A multi-mineral intervention to improve disease-related and mechanistic biomarkers in ulcerative colitis patients: results from a randomized trial. *PLoS One.* (2025) 20:e0337408. 10.1371/journal.pone.0337408 41359652 PMC12685183

[26] Adey W McKibbin D. Studies on the maerl species *Phymatolithon calcareum* (Pallas) nov. comb. and *Lithothamnium coralloides* Crouan in the Ria de Vigo. *Bot Mar.* (1970) 13:100–6. 10.1515/botm.1970.13.2.100 31755547

[27] Aslam MN Bassis CM Bergin IL Knuver K Zick SM Sen A et al. A Calcium-rich multimineral intervention to modulate colonic microbial communities and metabolomic profiles in humans: results from a 90-Day Trial. *Cancer Prev Res*. (2020) 13:101–16. 10.1158/1940-6207.CAPR-19-0325 31771942 PMC7528938

[28] Guyatt G Mitchell A Irvine EJ Singer J Williams N Goodacre R et al. A new measure of health status for clinical trials in inflammatory bowel disease. *Gastroenterology.* (1989) 96:804–10. 10.1016/0016-5085(89)90905-0 2644154

[29] National Cancer Institute. *Diet History Questionnaire, Version 3.0.* (2018). Available online at: https://epi.grants.cancer.gov/dhq3 (accessed December 30, 2025).

[30] Henriksen M Jahnsen J Lygren I Stray N Sauar J Vatn MH et al. C-reactive protein: a predictive factor and marker of inflammation in inflammatory bowel disease. Results from a prospective population-based study. *Gut*. (2008) 57:1518–23. 10.1136/gut.2007.146357 18566104

[31] Cappello M Randazzo C Bravatà I Licata A Peralta S Craxì A et al. Liver function test abnormalities in patients with inflammatory bowel diseases: a hospital-based survey. *Clin Med Insights Gastroenterol*. (2014) 7:25–31. 10.4137/CGast.S13125 24966712 PMC4069044

[32] van der Post S Jabbar KS Birchenough G Arike L Akhtar N Sjovall H et al. Structural weakening of the colonic mucus barrier is an early event in ulcerative colitis pathogenesis. *Gut*. (2019) 68:2142–51. 10.1136/gutjnl-2018-317571 30914450 PMC6872445

[33] Martini E Krug SM Siegmund B Neurath MF Becker C. Mend your fences: the epithelial barrier and its relationship with mucosal immunity in inflammatory bowel disease. *Cell Mol Gastroenterol Hepatol*. (2017) 4:33–46. 10.1016/j.jcmgh.2017.03.007 28560287 PMC5439240

[34] Wyatt J Vogelsang H Hübl W Waldhöer T Lochs H. Intestinal permeability and the prediction of relapse in Crohn’s disease. *Lancet*. (1993) 341:1437–9. 10.1016/0140-6736(93)90882-h 8099141

[35] Secondulfo M de Magistris L Fiandra R Caserta L Belletta M Tartaglione MT et al. Intestinal permeability in Crohn’s disease patients and their first degree relatives. *Dig Liver Dis*. (2001) 33:680–5. 10.1016/s1590-8658(01)80045-1 11785714

[36] Ménard S Cerf-Bensussan N Heyman M. Multiple facets of intestinal permeability and epithelial handling of dietary antigens. *Mucosal Immunol*. (2010) 3:247–59. 10.1038/mi.2010.5 20404811

[37] Noiraud N Maurousset L Lemoine R. Identification of a mannitol transporter, AgMaT1, in celery phloem. *Plant Cell*. (2001) 13:695–705. 10.1105/tpc.13.3.695 11251106 PMC135512

[38] Choe M Min H Park YH Kim YR Woo JS Seok YJ. Structural insight into glucose repression of the mannitol operon. *Sci Rep*. (2019) 9:13930. 10.1038/s41598-019-50249-2 31558743 PMC6763467

[39] O’Neill WC. Physiological significance of volume-regulatory transporters. *Am J Physiol*. (1999) 276:C995–1011. 10.1152/ajpcell.1999.276.5.C995 10329946

[40] Versantvoort CH Ondrewater RC Duizer E Van de Sandt JJ Gilde AJ Groten JP. Monolayers of IEC-18 cells as an in vitro model for screening the passive transcellular and paracellular transport across the intestinal barrier: comparison of active and passive transport with the human colon carcinoma Caco-2 cell line. *Environ Toxicol Pharmacol*. (2002) 11:335–44. 10.1016/s1382-6689(01)00122-3 21782616

[41] Fawley J Gourlay DM. Intestinal alkaline phosphatase: a summary of its role in clinical disease. *J Surg Res*. (2016) 202:225–34. 10.1016/j.jss.2015.12.008 27083970 PMC4834149

[42] Kühn F Adiliaghdam F Cavallaro PM Hamarneh SR Tsurumi A Hoda RS et al. Intestinal alkaline phosphatase targets the gut barrier to prevent aging. *JCI Insight*. (2020) 5:e134049. 10.1172/jci.insight.134049 32213701 PMC7213802

[43] Liu W Hu D Huo H Zhang W Adiliaghdam F Morrison S et al. Intestinal alkaline phosphatase regulates tight junction protein levels. *J Am Coll Surg*. (2016) 222:1009–17. 10.1016/j.jamcollsurg.2015.12.006 27106638 PMC5684582

[44] Bilski J Mazur-Bialy A Wojcik D Zahradnik-Bilska J Brzozowski B Magierowski M et al. The role of intestinal alkaline phosphatase in inflammatory disorders of gastrointestinal tract. *Mediators Inflamm*. (2017) 2017:9074601. 10.1155/2017/9074601 28316376 PMC5339520

[45] Mertz A Nguyen NA Katsanos KH Kwok RM. Primary sclerosing cholangitis and inflammatory bowel disease comorbidity: an update of the evidence. *Ann Gastroenterol*. (2019) 32:124–33. 10.20524/aog.2019.0344 30837784 PMC6394256

[46] Rupp C Rössler A Halibasic E Sauer P Weiss KH Friedrich K et al. Reduction in alkaline phosphatase is associated with longer survival in primary sclerosing cholangitis, independent of dominant stenosis. *Aliment Pharmacol Ther*. (2014) 40:1292–301. 10.1111/apt.12979 25316001

[47] Schicho R Shaykhutdinov R Ngo J Nazyrova A Schneider C Panaccione R et al. Quantitative metabolomic profiling of serum, plasma, and urine by (1)H NMR spectroscopy discriminates between patients with inflammatory bowel disease and healthy individuals. *J Proteome Res*. (2012) 11:3344–57. 10.1021/pr300139q 22574726 PMC3558013

[48] Laker MF Bull HJ Menzies IS. Evaluation of mannitol for use as a probe marker of gastrointestinal permeability in man. *Eur J Clin Invest*. (1982) 12:485–91. 10.1111/j.1365-2362.1982.tb02230.x 6818037

[49] Khoshbin K Khanna L Maselli D Atieh J Breen-Lyles M Arndt K et al. Development and validation of test for “Leaky Gut” small intestinal and colonic permeability using sugars in healthy adults. *Gastroenterology.* (2021) 161:463–475.e13. 10.1053/j.gastro.2021.04.020 33865841 PMC8328885

[50] Grover M Camilleri M Hines J Burton D Ryks M Wadhwa A et al. (13) C mannitol as a novel biomarker for measurement of intestinal permeability. *Neurogastroenterol Motil*. (2016) 28:1114–9. 10.1111/nmo.12802 26914765 PMC4920698

[51] Smecuol E Bai JC Vazquez H Kogan Z Cabanne A Niveloni S et al. Gastrointestinal permeability in celiac disease. *Gastroenterology*. (1997) 112:1129–36. 10.1016/s0016-5085(97)70123-9 9097995

[52] Cobden I Dickinson RJ Rothwell J Axon AT. Intestinal permeability assessed by excretion ratios of two molecules: results in coeliac disease. *Br Med J*. (1978) 2:1060. 10.1136/bmj.2.6144.1060 709218 PMC1608147

[53] Camilleri M Lasch K Zhou W. Irritable bowel syndrome: methods, mechanisms, and pathophysiology. The confluence of increased permeability, inflammation, and pain in irritable bowel syndrome. *Am J Physiol Gastrointest Liver Physiol*. (2012) 303:G775–85. 10.1152/ajpgi.00155.2012 22837345

[54] Szabo G. Gut-liver axis in alcoholic liver disease. *Gastroenterology*. (2015) 148:30–6. 10.1053/j.gastro.2014.10.042 25447847 PMC4274189

[55] Giorgio V Miele L Principessa L Ferretti F Villa MP Negro V et al. Intestinal permeability is increased in children with non-alcoholic fatty liver disease, and correlates with liver disease severity. *Dig Liver Dis*. (2014) 46:556–60. 10.1016/j.dld.2014.02.010 24631029

[56] van Elburg RM Uil JJ van Aalderen WM Mulder CJ Heymans HS. Intestinal permeability in exocrine pancreatic insufficiency due to cystic fibrosis or chronic pancreatitis. *Pediatr Res*. (1996) 39:985–91. 10.1203/00006450-199606000-00010 8725259

[57] Kowalski K Mulak A. Brain-gut-microbiota axis in Alzheimer’s Disease. *J Neurogastroenterol Motil*. (2019) 25:48–60. 10.5056/jnm18087 30646475 PMC6326209

[58] Forsyth CB Shannon KM Kordower JH Voigt RM Shaikh M Jaglin JA et al. Increased intestinal permeability correlates with sigmoid mucosa alpha-synuclein staining and endotoxin exposure markers in early Parkinson’s disease. *PLoS One*. (2011) 6:e28032. 10.1371/journal.pone.0028032 22145021 PMC3228722

[59] Rowin J Xia Y Jung B Sun J. Gut inflammation and dysbiosis in human motor neuron disease. *Physiol Rep*. (2017) 5:e13443. 10.14814/phy2.13443 28947596 PMC5617930

[60] Stevens BR Goel R Seungbum K Richards EM Holbert RC Pepine CJ et al. Increased human intestinal barrier permeability plasma biomarkers zonulin and FABP2 correlated with plasma LPS and altered gut microbiome in anxiety or depression. *Gut*. (2018) 67:1555–7. 10.1136/gutjnl-2017-314759 28814485 PMC5851874

[61] Tyszka M Biliński J Basak GW. Advances in intestinal barrier preservation and restoration in the allogeneic hematopoietic cell transplantation setting. *J Clin Med*. (2021) 10:2508. 10.3390/jcm10112508 34204044 PMC8201017

