# Supplementary material for: A multi-mineral intervention is associated with improved intestinal permeability in patients with ulcerative colitis: results from a pilot trial
Source: Front Med (Lausanne). 2026 Jun 22;13:1805900. doi: 10.3389/fmed.2026.1805900 (PMC13333513; doi:10.3389/fmed.2026.1805900)
Supplement: Supplementary file 1 [file Table_1.docx]

| **Supplementary Table 1: Elemental Composition and Daily Intake of Aquamin^®^ TG** | | | | | |
| --- | --- | --- | --- | --- | --- |
|  |  |  |  |  |  |
| Element | μg/day | Element | μg/day | Element | μg/day |
| Calcium | 800000 | Chromium | 7.7 | Erbium | 0.3 |
| Carbon | 317949 | Ruthenium | 5.2 | Gadolinium | 0.3 |
| Magnesium | 66154 | Lanthanum | 5.1 | Gallium | 0.3 |
| Sodium | 12528 | Cobalt | 4.8 | Hafnium | 0.3 |
| Sulfur | 8264 | Beryllium | 4.6 | Iodine | 0.3 |
| Strontium | 6033 | Nickel | 4.6 | Praseodymium | 0.3 |
| Chloride | 5903 | Scandium | 4.4 | Samarium | 0.3 |
| Iron | 1562 | Arsenic | 3.1 | Ytterbium | 0.2 |
| Potassium | 513 | Cerium | 2 | Europium | 0.1 |
| Silicon | 233 | Cadmium | 1.7 | Holmium | 0.1 |
| Phosphorous | 200 | Neodymium | 1.2 | Lutetium | 0.1 |
| Aluminum | 189 | Palladium | 1.2 | Rubidium | 0.06 |
| Manganese | 111 | Rhodium | 1 | Terbium | 0.06 |
| Boron | 88 | Antimony | <1.3 | Thulium | 0.04 |
| Titanium | 82 | Bismuth | <1.3 | Tantalum | 0.02 |
| Niobium | 31 | Gold | <1.3 | Rhenium | 0.01 |
| Barium | 19 | Lithium | <1.3 | Cesium | 0.005 |
| Zirconium | 14 | Molybdenum | <1.3 | Osmium | 0.003 |
| Thorium | 11 | Selenium | <1.3 | Germanium | <0.003 |
| Vanadium | 9.4 | Tellurium | <1.3 | Indium | <0.003 |
| Yttrium | 9.4 | Thallium | <1.3 | Iridium | <0.003 |
| Silver | 9.1 | Tungsten | <1.3 | Mercury | <0.003 |
| Fluoride | 9 | Tin | 0.6 | Platinum | <0.003 |
| Copper | 8.4 | Dysprosium | 0.4 |  |  |
| Zinc | 7.8 | Lead | 0.4 |  |  |

Values are reported based on the daily intake of Aquamin^®^ TG standardized to deliver 800 mg of calcium. The trace mineral composition of Aquamin^®^ TG was analyzed in 2018 by an independent laboratory (Advanced Laboratories, Inc., Salt Lake City) for Marigot Limited (Ireland). Individual trace elements are listed in order of their abundance, as determined primarily by Inductively Coupled Plasma Optical Emission Spectroscopy (ICP-OES), with carbon quantified by ASTM D-1552, chloride and iodine by titration, and fluoride by AOAC 939.11.
