# Supplementary material for: A multi-mineral intervention is associated with improved intestinal permeability in patients with ulcerative colitis: results from a pilot trial
Source: Front Med (Lausanne). 2026 Jun 22;13:1805900. doi: 10.3389/fmed.2026.1805900 (PMC13333513; doi:10.3389/fmed.2026.1805900)
Supplement: Supplementary file 2 [file Table_2.docx]

| **Supplementary Table 2. Health-related quality of life assessment (IBDQ) scoring** | | | |
| --- | --- | --- | --- |
|  |  |  |  |
| IBDQ Measures | Normal Range | Pre | Post |
| T-score (Total) | *32–224* | 191.6 ± 15.3 | 194.3 ± 20.5 |
| Bowel symptoms score | *10–70* | 61.4 ± 5.9 | 62.3 ± 7.2 |
| Systemic symptoms score | *5–35* | 27.5 ± 4.2 | 28.1 ± 4.7 |
| Emotional function score | *12–84* | 68.6 ± 7.0 | 69.8 ± 7.7 |
| Social function score | *5–35* | 34.1 ± 1.1 | 34.1 ± 2.1 |

Values represent Mean ± Standard Deviation for each respective measure; n = 8 subjects. The observed baseline IBDQ total score range was 172–200. A total IBDQ score of ≥ 170 is commonly used as a cutoff for clinical remission. The Inflammatory Bowel Disease Questionnaire (IBDQ) is a 32-item, patient-reported instrument assessing four domains: bowel symptoms, systemic symptoms, emotional function, and social function, using 7-point Likert-type scales. Higher scores indicate better quality of life; domain scores correspond to their specific item ranges.
