# Supplementary material for: A multi-mineral intervention is associated with improved intestinal permeability in patients with ulcerative colitis: results from a pilot trial
Source: Front Med (Lausanne). 2026 Jun 22;13:1805900. doi: 10.3389/fmed.2026.1805900 (PMC13333513; doi:10.3389/fmed.2026.1805900)
Supplement: Supplementary file 3 [file Table_3.docx]

| **Supplementary Table 3.** **Demographic Data for Subjects Enrolled in the 90-Day Study** | | | | | | | | | |
| --- | --- | --- | --- | --- | --- | --- | --- | --- | --- |
| **A. Study cohort demographics:** |  |  |  |  | |  | |  | |
|  | **Female** | | **Male** | | | **Both Genders** | | | |
| **Ethnic Category** | N | % | N | % | | Total | | % | |
| Hispanic or Latino | 1 | 3.23% | 2 | 6.45% | | 3 | | 9.68% | |
| Not Hispanic or Latino | 14 | 45.16% | 13 | 41.94% | | 27 | | 87.10% | |
| Unknown | 0 | 0.00% | 0 | 0.00% | | 0 | | 0.00% | |
| Prefer not to answer | 1 | 3.23% | 0 | 0.00% | | 1 | | 3.23% | |
| **Total** | **16** | **51.61%** | **15** | **48.39%** | | **31** | | **100.00%** | |
| **Racial Category**  *(single category per participant)* | N | % | N | % | | Total | | % | |
| American Indian/Alaska Native | 1 | 3.12% | 0 | 0.00% | | 1 | | 3.12% | |
| Asian | 2 | 6.25% | 1 | 3.12% | | 3 | | 9.38% | |
| Native Hawaiian or Pacific Islander | 0 | 0.00% | 0 | 0.00% | | 0 | | 0.00% | |
| Black or African American | 0 | 0.00% | 2^a^ | 6.25% | | 2^a^ | | 6.25% | |
| White | 13 | 40.63% | 13^a^ | 40.63% | | 26^a^ | | 81.25% | |
| Other | 0 | 0.00% | 0 | 0.00% | | 0 | | 0.00% | |
| Unknown | 0 | 0.00% | 0 | 0.00% | | 0 | | 0.00% | |
| **Total** | **16** | **50.00%** | **16**^a^ | **50.00%** | | **32**^a^ | | **100.00%** | |
| **Age at Enrollment Category** | N | % | N | % | | Total | | % | |
| 18 - 21 years | 4 | 12.90% | 1 | 3.23% | | 5 | | 16.13% | |
| 22 - 29 years | 3 | 9.68% | 3 | 9.68% | | 6 | | 19.35% | |
| 30 - 39 years | 3 | 9.68% | 5 | 16.13% | | 8 | | 25.81% | |
| 40 - 49 years | 4 | 12.90% | 3 | 9.68% | | 7 | | 22.58% | |
| 50 - 59 years | 1 | 3.23% | 2 | 6.45% | | 3 | | 9.68% | |
| 60 - 69 years | 1 | 3.23% | 1 | 3.23% | | 2 | | 6.45% | |
| 70 - 79 years | 0 | 0.00% | 0 | 0.00% | | 0 | | 0.00% | |
| > 80 years | 0 | 0.00% | 0 | 0.00% | | 0 | | 0.00% | |
| **Total** | **16** | **51.61%** | **15** | **48.39%** | | **31** | | **100.00%** | |
| **^a^** One healthy male subject selected both White and African American racial categories | | | | | | | | | |
| **B. Gender and Age:** |  |  |  |  | |  | |  | |
|  | Gender | Age (Y) |  |  | |  | |  | |
| Healthy (n=19) | M:9 / F:10 | 31.7±12.0 |  |  | |  | |  | |
| UC (n=12) | M:6 / F:6 | 41.9±12.7 |  |  | |  | |  | |
| Age presented in years at the start of study participation. | | | | | | | | | |
|  |  |  |  |  | |  | |  | |
| **C. Body Mass Index:** | BMI - Weight/Height^2^ (Kg/m^2^) | | | |  | |  | |  |
|  | Pre | Post |  |  | |  | |  | |
| Healthy (n=12) | 24.3±4.0 | 24.6±4.4 |  |  | |  | |  | |
| UC (n=8) | 26.5±5.7 | 27.6±5.3 |  |  | |  | |  | |
|  |  |  |  |  | |  | |  | |
