# Supplementary material for: A multi-mineral intervention is associated with improved intestinal permeability in patients with ulcerative colitis: results from a pilot trial
Source: Front Med (Lausanne). 2026 Jun 22;13:1805900. doi: 10.3389/fmed.2026.1805900 (PMC13333513; doi:10.3389/fmed.2026.1805900)
Supplement: Supplementary file 4 [file Table_4.docx]

**Supplementary Table 4. Summary of Average Mineral Intake and Recommended Levels**

|  | Healthy Subjects | | | |  | Subjects with UC | | | |
| --- | --- | --- | --- | --- | --- | --- | --- | --- | --- |
| ***Nutrient (Unit)*** | Eaten | Suggested Target | Min | Max |  | Eaten | Suggested Target | Min | Max |
| **Calcium (mg)** | 1089.0 ± 526.4 | 1120.0 ± 164.3 | 384 | 1775 |  | 1169.0 ± 415.1 | 1028.6 ± 75.6 | 565 | 1811 |
| **Copper (mg)** | 1.3 ± 0.6 | 0.9 ± 0.0 | 0.7 | 2 |  | 1.5 ± 0.4 | 0.9 ± 0.0 | 0.7 | 2 |
| **Iron (mg)** | 11.8 ± 6.0 | 12.8 ± 4.5 | 5.7 | 20 |  | 13.6 ± 3.2 | 13.7 ± 5.3 | 8.3 | 18 |
| **Magnesium (mg)** | 293.0 ± 110.9 | 372.0 ± 39.0 | 145 | 420 |  | 377.1 ± 119.6 | 345.7 ± 44.3 | 191 | 530 |
| **Phosphorus (mg)** | 1173.4 ± 475.8 | 920.0 ± 301.2 | 597 | 1890 |  | 1436.6 ± 417.0 | 700.0 ± 0.0 | 705 | 1918 |
| **Potassium (mg)** | 2325.4 ± 1126.2 | 4700.0 ± 0.0 | 1195 | 3916 |  | 2701.9 ± 686.1 | 4700.0 ± 0.0 | 1773 | 3796 |
| **Selenium (mcg)** | 103.0 ± 51.2 | 55.0 ± 0.0 | 48 | 187 |  | 79.7 ± 26.0 | 55.0 ± 0.0 | 53 | 128 |
| **Zinc (mg)** | 9.3 ± 3.6 | 9.6 ± 1.3 | 5.4 | 15 |  | 11.2 ± 2.9 | 8.9 ± 1.5 | 5.5 | 14 |
| **Vitamin D (mcg)** | 2.2 ± 0.7 | 15.0 ± 0.0 | 1.6 | 3 |  | 5.8 ± 3.8 | 15.0 ± 0.0 | 1.9 | 12 |

Participants (n = 5 for healthy subjects and n = 7 for UC subjects) reported their food and beverage intake, including quantities and frequency, over the past year using the NIH Diet History Questionnaire III (DHQ3), developed by the National Cancer Institute (NCI). These responses were used to calculate average daily intakes of nutrients and food groups. Average intakes were then compared to current dietary guidelines based on age and sex, as outlined in the Dietary Guidelines for Americans¹. The table presents a subset of mineral intakes calculated using the DHQ3 database^a^, expressed as means and standard deviations. These are the only minerals detected by DHQ3.

Note: Vitamin D intake in DHQ3 is calculated from food, beverage, and supplement data only. Sun exposure and cutaneous vitamin D synthesis are not assessed by DHQ3.

^1^Dietary Guidelines for Americans. <https://www.dietaryguidelines.gov/resources/2020-2025-dietary-guidelines-online-materials>

^a^Mineral values were calculated using the Diet History Questionnaire version 3 (DHQ3) database.
