# Supplementary material for: A multi-mineral intervention is associated with improved intestinal permeability in patients with ulcerative colitis: results from a pilot trial
Source: Front Med (Lausanne). 2026 Jun 22;13:1805900. doi: 10.3389/fmed.2026.1805900 (PMC13333513; doi:10.3389/fmed.2026.1805900)
Supplement: Supplementary file 5 [file Table_5.docx]

| **Supplementary Table 5. Treatment-Related Reportable Adverse Events** | | |
| --- | --- | --- |
| **Event** | ***Healthy*** | ***UC*** |
| Number of subjects received intervention | 13 | 9 |
| Number of subjects reporting events | 7 ^a^ | 6 |
| Total number of adverse events | 21 | 10 |
| Bone graft surgery for dental implant | 0 | 1 (1) |
| Dizziness (light headedness) | 1 (1) | 0 |
| Fatigue | 0 | 1 (1) |
| Joint pain | 0 | 1 (1) |
| Hepatobiliary disorders (mild elevated LFTS) | 2 (2) | 0 |
| Gilberts disease | 1 (1) | 0 |
| Tonsil stones | 1 (1) | 0 |
| Gastrointestinal events |  |  |
| *Nausea* | 2 (2) | 0 |
| *Flatulence (or gas)* | 7 (5) | 1 (1) |
| *Abdominal discomfort (with bloating)* | 3 (3) | 3 (3) |
| *Constipation* | 2 (2) | 1 (1) |
| *Minor blood in stool with lower abdominal ache* | 0 | 1 (1) |
| Mannitol/Lactulose-related AEs: |  |  |
| Diarrhea (loose stool) | 0 | 1 (1) |
| OCD triggered by mannitol/lactulose intake & urine collection ^a^ | 1 (1) | 0 |
| Migraine headache | 1 (1) | 0 |

Numbers in parentheses represent subjects who experienced an adverse event. The study physician (DKT) determined that most adverse events were unrelated to the study and attributed them to pre-existing conditions or concomitant medications, except for those affecting the gastrointestinal system. Some events were reported multiple times or together (for example, gas and discomfort), but these are listed separately.

Mannitol/Lactulose-related events were not related to the study agent (Aquamin^®^) as these events were reported prior to Aquamin^®^ ingestion.

^a^ Subject with OCD (obsessive-compulsive disorder) symptoms; withdrew from the study after the initial urine collection procedure.
