# Supplementary figures and images for: A multi-mineral intervention is associated with improved intestinal permeability in patients with ulcerative colitis: results from a pilot trial
Source: Front Med (Lausanne). 2026 Jun 22;13:1805900. doi: 10.3389/fmed.2026.1805900 (PMC13333513; doi:10.3389/fmed.2026.1805900)

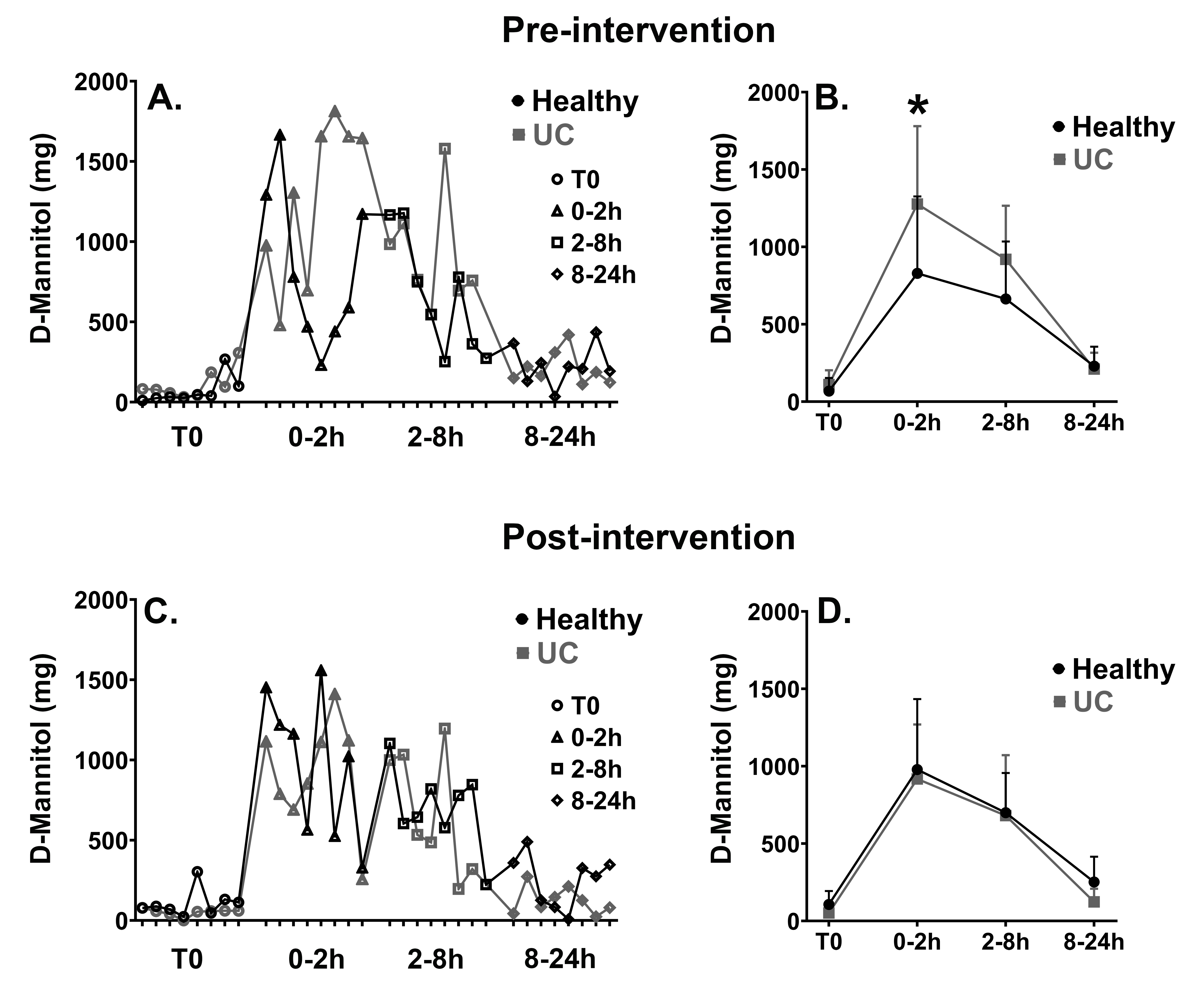

Supplement: Supplementary Figure 1 — (A,B) Pre-intervention (Day 0). (C,D) Post-intervention (Day 90) urinary D-mannitol excretion in healthy subjects and patients with UC. Pre- and post-intervention urinary D-mannitol (mg) recovered in sequential urine collections (T0, 0–2 h, 2–8 h, and 8–24 h) following an oral mannitol challenge are shown for healthy subjects versus subjects with UC in spaghetti plots. *P < 0.05 for the UC vs healthy comparison of mannitol excretion at 0–2 h prior to intervention. [file Image_1.tif]

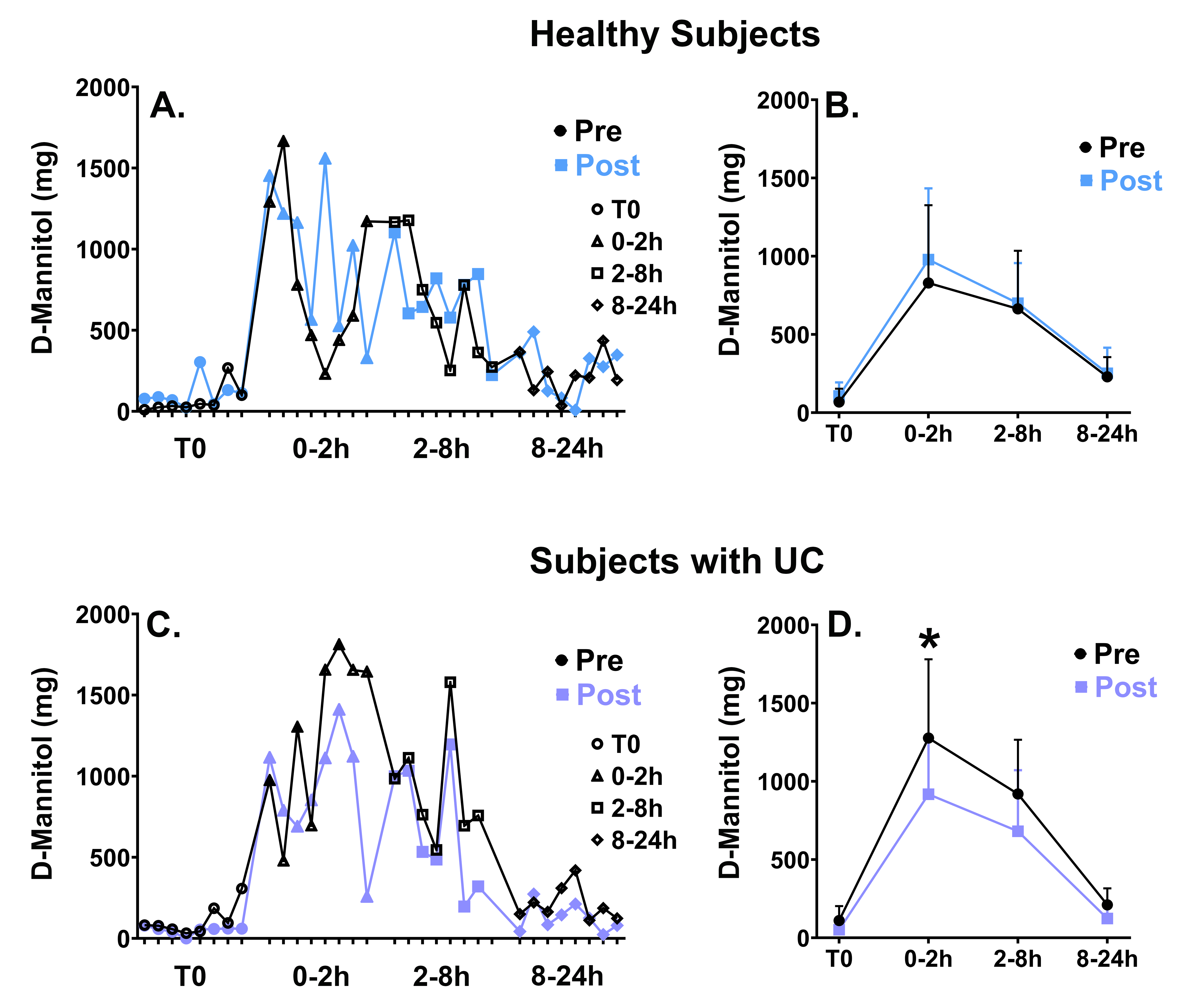

Supplement: Supplementary Figure 2 — Effect of Aquamin® intervention on urinary D-mannitol excretion in healthy subjects (A,B) and UC subjects (C,D) at baseline (Day 0; Pre-intervention) and Day 90 (Post-intervention). Urinary D-mannitol (mg) in each collection interval (T0, 0–2 h, 2–8 h, and 8–24 h) is shown at baseline (Pre-intervention) and after 90 days (Post-intervention) in spaghetti plots. * indicates P < 0.05 for the indicated pre–post comparisons in UC subjects; the pre-post difference was significant at 0–2 h. [file Image_2.tif]
